# Improvement of cDNA TRAP Display via Optimization of Puromycin Linker Design for Enhanced Discovery of Antibody‐Like Proteins

**DOI:** 10.1002/cbic.70375

**Published:** 2026-05-26

**Authors:** Haruto Kosugi, Hiroki Nakanishi, Gosuke Hayashi, Hiroshi Murakami

**Affiliations:** ^1^ Department of Biomolecular Engineering, Graduate School of Engineering Nagoya University Nagoya Japan; ^2^ Institute of Nano‐Life‐Systems, Institutes of Innovation for Future Society Nagoya University Nagoya Japan; ^3^ Research Institute for Quantum and Chemical Innovation, Institutes of Innovation for Future Society Nagoya University Nagoya Japan

**Keywords:** cDNA TRAP display, directed evolution, in vitro selection, oligonucleotides, protein engineering

## Abstract

cDNA TRAP display is a high‐speed in vitro selection method for obtaining antibody‐like proteins (ALPs) with desired functions, enabling selection even under harsh conditions. However, since its development, this method has not been applied to practical selection because of the low formation efficiency of the ALP–puromycin linker/mRNA complex (display efficiency), which is a key determinant of library diversity. In this study, we aimed to improve display efficiency by optimizing the puromycin linker design. Specifically, we evaluated modifications to the nucleotides at the 5′ end and attachment position of the puromycin moiety at the 3′ end, which resulted in a 1.8‐fold increase in display efficiency. These findings provide insights into enhancing the performance of cDNA TRAP display and are expected to facilitate future ALP discovery.

## Graphical abstract

The formation efficiency of the antibody‐like protein–puromycin linker/mRNA complex (display efficiency) has been improved in cDNA TRAP display. We optimized the puromycin linker design by modifying the structure and sequence at the 5’ end and the position of the puromycin moiety, achieving a 1.8‐fold increase in display efficiency.

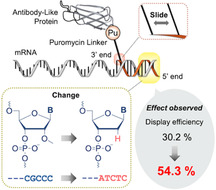

## 1 Introduction

In vitro selection techniques have been widely used to obtain functional peptides and antibody‐like proteins (ALPs). Among these techniques, mRNA display is one of the most widely used methods [1, 2]. In mRNA display, a puromycin moiety attached to the 3′ end of mRNA attacks the ribosome‐bound nascent polypeptide during translation in a cell‐free translation system, resulting in the formation of a covalent polypeptide–puromycin–mRNA complex (“–” represents a covalent bond). One of the major advantages of mRNA display is its ability to provide extremely large library diversity exceeding 10^13^. Recent studies have demonstrated a correlation between library diversity and the binding affinity of selected ligands [3, 4], suggesting that the high library diversity achievable by mRNA display is a critical factor for efficient ligand selection. However, a major drawback of mRNA display is that preparation of the puromycin–mRNA conjugate requires multiple laborious steps, making the overall process time‐consuming [5].

To address this issue, we previously developed TRAP (transcription–translation coupled with association of puromycin linker) display [6], a simplified and accelerated version of mRNA display (Fig. S1a and Fig. S1b). With this method, a polypeptide–puromycin linker (PuL)/mRNA complex can be generated within 30 min by simply adding a DNA library to a reconstituted cell‐free translation system (“/” represents a noncovalent bond). Using TRAP display, we have successfully selected macrocyclic peptides [7] and ALPs [8, 9] against a variety of targets. Notably, the sequence of a monobody with neutralizing activity against the receptor‐binding domain of the SARS‐CoV‐2 spike protein was identified in only 4 days [8]. We also recently optimized the sequence of the mRNA used in TRAP display, achieving library diversity (10^14^) exceeding that of mRNA display [10].

mRNA display and TRAP display are powerful techniques for obtaining ALPs against a wide range of targets. However, because ALPs are indirectly linked to their corresponding cDNA via mRNA, these techniques cannot be performed in the presence of RNases (ribonucleases). In addition, the noncovalent linkage between cDNA and mRNA limits the tolerance to harsh conditions, including prolonged washing, high temperatures, and strong bases.

An alternative approach that can address these limitations is cDNA display [11, 12, 13]. Unlike conventional approaches, this technology forms a polypeptide–PuL–cDNA complex, which reduces the vulnerability associated with mRNA and enhances complex stability [14]. This has enabled selection in the presence of RNases, facilitating the discovery of ligands with high activity and stability in serum [15] and the selection targeting live cells [16, 17]. In addition, selection under basic conditions has also been reported [18].

Following this strategy, we developed cDNA TRAP display (Fig. S1c and Fig. S2) [19], which enables more rapid preparation of ALP–PuL–cDNA complexes compared with cDNA display. However, since its development, cDNA TRAP display has not been applied to practical selection due to the low formation efficiency of the ALP–PuL/mRNA complex (hereafter referred to as “display efficiency,” as it represents the efficiency with which an ALP is displayed by its mRNA), which determines the achievable library diversity. Moreover, no efforts have been reported to improve the display efficiency of cDNA TRAP display. In this study, we focused on improving display efficiency through optimization of the puromycin linker design. First, we modified the nucleotides at the 5′ end and analyzed how their structure and sequence affect display efficiency. Next, we evaluated whether the attachment position of the puromycin moiety influences display efficiency. These findings provide insights into enhancing the performance of cDNA TRAP display and may facilitate future ligand discovery efforts.

## 2 Results and Discussion

### 2.1 Improvement of Display Efficiency by Using the Optimized mRNA Sequence

**FIGURE 1:**
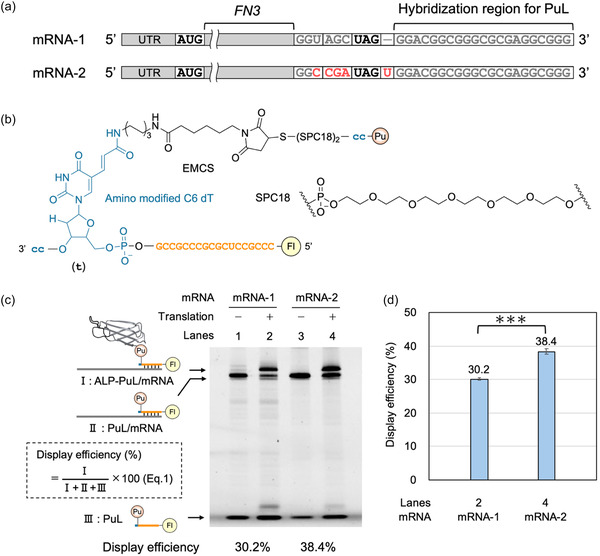
Improvement of display efficiency in cDNA TRAP display through mRNA sequence engineering. (a) Schematic representation of the mRNA sequences encoding ALPs: mRNA‐1, with the original sequence; and mRNA‐2, with the optimized sequence near the UAG nonsense codon, which has been shown to improve the display efficiency of original TRAP display. (b) The detailed structure of the PuL, Fl‐CCCGC‐t[Pu]cc. DNA and 2′‐OMe RNA are shown in blue and orange, respectively. The 5′ end of the PuL was labeled with fluorescein (Fl) to visualize the PuL in gels. Synthesis scheme of this PuL is shown in Fig. S3. EMCS and SPC18 represent N‐(6‐maleimidocaproyloxy) sulfosuccinimide and spacer 18, respectively. (c) Urea SDS‐PAGE analysis of the display efficiencies of mRNAs with the original or the optimized sequence. Display efficiencies were calculated using (Equation (1)). (d) Quantification of display efficiency based on the urea SDS‐PAGE analysis shown in Figure 1c. Error bars represent the standard deviation of triplicate experiments. SDS‐PAGE = SDS‐polyacrylamide gel electrophoresis.

Inspired by a previous study showing that engineering the mRNA sequence near the UAG nonsense codon can improve the display efficiency of original TRAP display [10], we applied the optimized mRNA sequence to cDNA TRAP display (Figure 1a). mRNAs encoding the wild‐type monobody (the 10th human fibronectin type III domain; FN3) with either the original (mRNA‐1) or optimized sequence (mRNA‐2) were annealed to the PuL (Fl‐CCCGC‐t[Pu]cc; Fl, fluorescein; Pu, puromycin; uppercase letters, 2^′^‐OMe RNA; lowercase letters, DNA; Figure 1b) and added to the translation reaction mixture. After incubation at 37°C for 30 min, display efficiency was analyzed by urea SDS‐polyacrylamide gel electrophoresis (SDS‐PAGE). As expected, mRNA‐2 exhibited increased display efficiency compared with mRNA‐1 (Figure 1c, Lane 2 vs Lane 4; Figure 1d, 30.2% vs 38.4%). This optimized sequence has been proposed to enhance puromycin conjugation to the displayed protein through suppression of UAG codon readthrough in original TRAP display [10], and our results suggest that this effect is also applicable to cDNA TRAP display.

### 2.2 Comparison of Display Efficiency between the cDNA TRAP and the cDNA Display‐Based System

Although we successfully improved the display efficiency of cDNA TRAP display through mRNA sequence engineering, this method was still considered to have an inherent limitation. Because the PuL is annealed to mRNA without a covalent linkage, dissociation of the PuL/mRNA complex during translation can limit display efficiency. In contrast, in conventional cDNA display, the PuL and mRNA are ligated prior to translation [20, 21, 22, 23], allowing formation of a more stable PuL–mRNA complex. Therefore, comparison of display efficiency between the cDNA TRAP system and the ligation‐containing cDNA display‐based system allows evaluation of the performance of cDNA TRAP display as a selection method.

**FIGURE 2:**
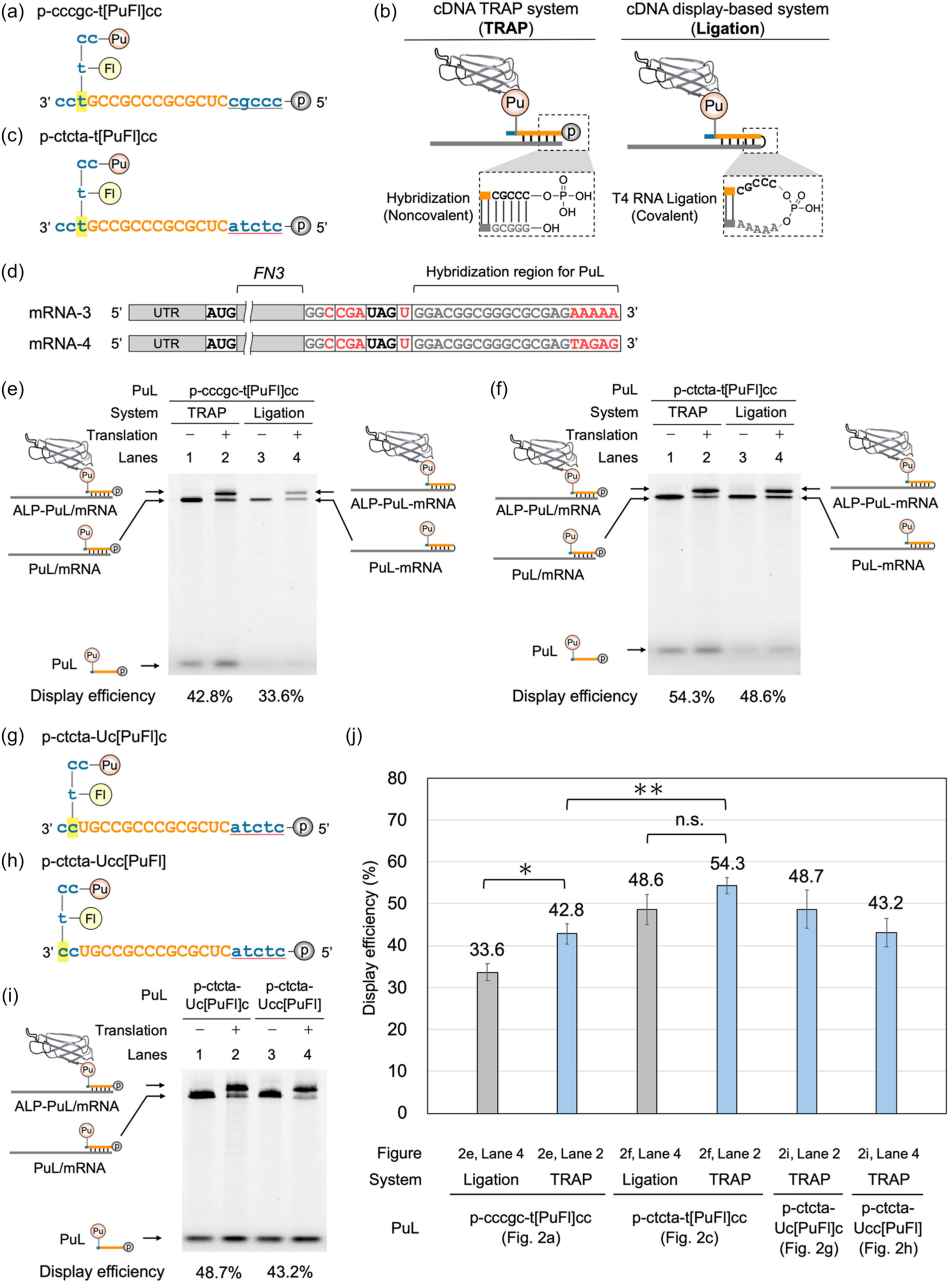
Improvement of display efficiency in cDNA TRAP display through optimization of PuL design. (a) Structure of p‐cccgc‐t[PuFl]cc. DNA and 2′‐OMe RNA are shown in blue and orange, respectively. The detailed structure is shown in Fig. S6a. (b) Schematic representation of displayed ALP complexes formed in the cDNA TRAP system and the ligation‐containing cDNA display‐based system. In the cDNA TRAP system (TRAP), the PuL is annealed to the corresponding mRNA without ligation. In the cDNA display‐based system (Ligation), the PuL and the mRNA with an AAAAA sequence at the 5′ end are covalently linked via a phosphodiester linkage. (c) Structure of p‐ctcta‐t[PuFl]cc. Color coding is the same as in Figure 2a. The detailed structure is shown in Fig. S6b. (d) Schematic representation of the sequences of mRNA‐3 and mRNA‐4. The 5‐mer nucleotide at the 3′ end of mRNA‐2 was modified. (e) Urea SDS‐PAGE analysis of display efficiencies using p‐cccgc‐t[PuFl]cc without or with ligation. (f) Urea SDS‐PAGE analysis of display efficiencies using p‐ctcta‐t[PuFl]cc without or with ligation. (g), (h) Structures of p‐ctcta‐Uc[PuFl]c and p‐ctcta‐Ucc[PuFl], respectively. Color coding is the same as in Figure 2a. The detailed structures are shown in Fig. S6c and Fig. S6d. (i) Urea SDS‐PAGE analysis of display efficiencies using p‐ctcta‐Uc[PuFl]c or p‐ctcta‐Ucc[PuFl]. (j) Quantification of display efficiency based on the urea SDS‐PAGE analyses shown in Figure 2e, Figure 2f, and Figure 2i. Error bars represent the standard deviation of triplicate experiments.

Accordingly, we synthesized a new PuL (p‐cccgc‐t[PuFl]cc; Figure 2a) through redesign of the original PuL by relocating the fluorescein moiety, introducing a 5′ phosphate, and replacing the 5‐mer 2′‐OMe RNA at the 5′ end with the corresponding DNA to perform RNA–DNA ligation via a phosphodiester linkage using T4 RNA ligase (Figure 2b) [6]. As the ligation acceptor, we prepared a new mRNA (mRNA‐3; Figure 2d) in which the 5‐mer nucleotide at the 3′ end (GGGCG) was replaced with adenosines (AAAAA). This design was based on a previous study, showing that a poly(A) sequence facilitates efficient ligation by T4 RNA ligase [24].

Following ligation between p‐cccgc‐t[PuFl]cc and mRNA‐3 (Fig. S4, Lane 3), the ligated complex was added to the translation reaction mixture, and the display efficiency was measured using the same method. As a result, the display efficiency achieved with ligation was comparable to that achieved without ligation described above (Figure 1c, Lane 4; Figure 1d, 38.4% vs Figure 2e, Lane 4; Figure 2j, 33.6%). Thus, by optimizing the mRNA sequence, we established a selection method that exhibits high display efficiency without requiring ligation. This is highly advantageous because ligation requires multiple procedures and cDNA TRAP display can eliminate this requirement.

Interestingly, when the display efficiency was measured using p‐cccgc‐t[PuFl]cc and its complementary mRNA‐2 without ligation, a slight increase was observed (Figure 2e, Lane 2; Figure 2j, 42.8%). In contrast, no improvement in display efficiency was observed for p‐CCCGC‐t[PuFl]cc, which retained 2′‐OMe RNA at the 5′ end (Fig. S5a and Fig. S5b, 37.4%), indicating that replacement of the 5‐mer nucleotide at the 5′ end from 2′‐OMe RNA to DNA contributes to the increase in display efficiency. Although the precise mechanism remains unclear, the 5′‐end 2′‐OMe RNA could hinder annealing to the mRNA through intramolecular secondary structure formation. By contrast, substitution of this region with DNA likely reduced such structural constraints, thereby resulting in improved display efficiency.

### 2.3 Optimization of Display Efficiency by Modifying the 5^′^‐End Sequence of the Puromycin Linker

Given the observed influence of the 5‐mer nucleotide at the 5′ end of the PuL on display efficiency, we next explored whether further modification of this region could yield additional improvements. One potential drawback of the original PuL is its high guanine and cytosine content. Although GC‐rich annealing regions are expected to form stable complementary strands, they may also promote the formation of undesired secondary structures [25]. To mitigate this possibility, the 5‐mer nucleotide at the 5′ end of p‐cccgc‐t[PuFl]cc was replaced with ctcta, thereby reducing the GC content and generating a new linker, p‐ctcta‐t[PuFl]cc (Figure 2c). Annealing this new PuL to the complementary mRNA (mRNA‐4; Figure 2d) resulted in an ≈11.5% increase in display efficiency compared with the original PuL (Figure 2e, Lane 2 vs Figure 2f, Lane 2; Figure 2j, 42.8% vs 54.3%). The reduction in GC content may have suppressed the formation of secondary structures in the PuL and/or mRNA that inhibit annealing, thereby contributing to the observed increase in display efficiency. Notably, even without ligation, this new PuL also showed display efficiency comparable to that achieved with ligation (Fig. S4, Lane 5 and Figure 2f, Lane 4; Figure 2j, 48.6%).

We also applied this effective 5′‐end PuL modification to original TRAP display. Similar to cDNA TRAP display, replacement of the 5‐mer nucleotide at the 5′ end from 2′‐OMe RNA to DNA, followed by further sequence alteration from cccgc to ctcta, significantly enhanced the display efficiency of original TRAP display (Fig. S9). These results demonstrate that the combination of the PuL 5′‐end structure and the mRNA 3′‐end sequence proposed in this study effectively enhances display efficiency in original TRAP display via the same mechanism as that observed in cDNA TRAP display.

### 2.4 Evaluation of the Effect of Puromycin Moiety Placement on Display Efficiency

Finally, we investigated the optimization at the 3′ end of the PuL. In the original design, the puromycin moiety was attached to the third nucleotide from the 3′ end of the PuL. We hypothesized that a shorter distance between the attachment position of the puromycin moiety and the ribosomal A‐site would facilitate puromycin incorporation and enhance conjugation efficiency, thereby improving display efficiency. Based on this hypothesis, two additional PuLs were prepared in which the puromycin moiety was attached to the second nucleotide (p‐ctcta‐Uc[PuFl]c; Figure 2g) or the first nucleotide (p‐ctcta‐Ucc[PuFl]; Figure 2h) from the 3′ end of the PuL.

Following annealing to mRNA‐4 and analysis under identical conditions, no significant improvement was observed with the newly designed PuLs, contrary to our hypothesis (Figure 2f, Lane 2 vs Figure 2i, Lanes 2 and 4; Figure 2j, 54.3% vs 48.7% or 43.2%). These results indicate that changing the attachment position of the puromycin moiety by one or two nucleotides does not significantly affect display efficiency.

### 2.5 Validation of Display Efficiency Using a Pulldown Assay

Overall, we achieved a 1.8‐fold improvement in display efficiency (Figure 1c, Lane 2; Figure 1d, 30.2% vs Figure 2f, Lane 2; Figure 2j, 54.3%). To further validate the reliability of the improvement in display efficiency determined by gel electrophoresis, we performed an independent pulldown‐based assay (Fig. S10a) using either the original mRNA/PuL pair or the optimized pair that exhibited the highest display efficiency.

Biotin‐Phe was introduced at the start codon using the Flexizyme system. After translation and reverse transcription, we first analyzed the display efficiency by gel electrophoresis, as changing the initiation amino acid could affect display efficiency. Although the absolute display efficiencies were lower when biotin‐Phe was used as the initiation amino acid compared with the canonical start codon—likely due to reduced translation initiation efficiency caused by incorporation of biotin‐Phe—the relative improvement (1.7‐fold) was consistent with the results described above (Fig. S10c and Fig. S10d).

The resulting biotin‐Phe–monobody–PuL–cDNA/mRNA complex was captured using streptavidin beads. The associated cDNA was then quantified by real‐time PCR. In this pulldown‐based assay, a similar trend was observed, with an ≈1.5‐fold improvement in display efficiency (Fig. S10b). These results further support that the improvement in display efficiency is reproducible in the pulldown experiment.

### 2.6 Comparison of Display Efficiencies of Each Puromycin Linker in the DNA‐Start System

**FIGURE 3:**
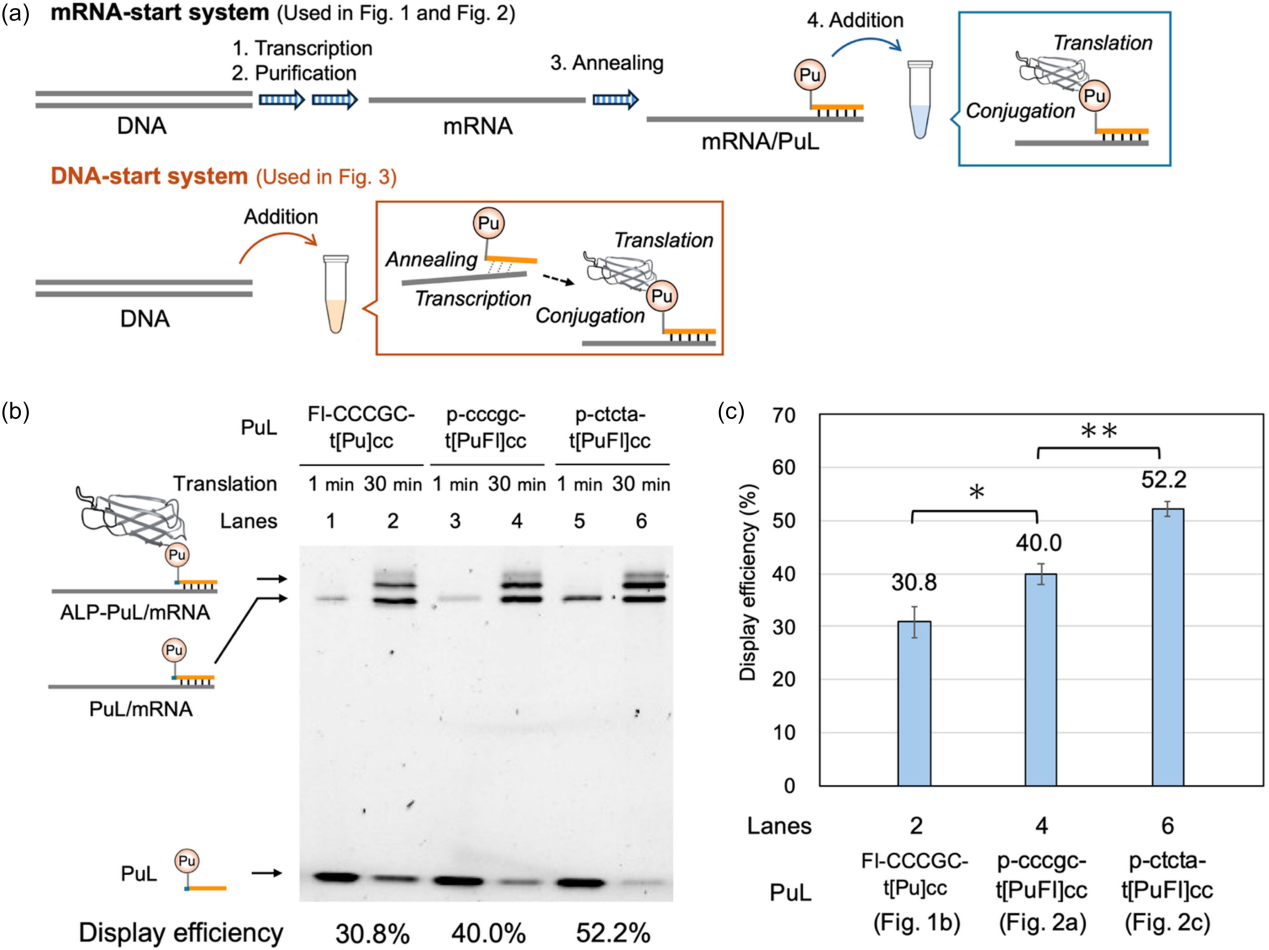
Improvement of the display efficiency of cDNA TRAP display in the DNA‐start system. (a) Schematic representation of the mRNA‐start and DNA‐start systems. In the mRNA‐start system, transcription, purification, and annealing of mRNA with the PuL are performed prior to translation. In contrast, in the DNA‐start system, simply adding DNA to a reconstituted cell‐free translation system allows transcription, annealing with the PuL, and translation to proceed sequentially, thereby eliminating multiple preparative steps. (b) Urea SDS‐PAGE analysis of display efficiencies of each PuL in the DNA‐start system. (c) Quantification of display efficiency based on the urea SDS‐PAGE analysis shown in Figure 3b. Error bars represent the standard deviation of triplicate experiments.

Up to this point, translation reactions were performed after annealing the puromycin linker to mRNA (mRNA‐start system). In contrast, as described above, cDNA TRAP display allows preparation of ALP–puromycin/mRNA complexes simply by adding DNA to a reconstituted cell‐free translation system (DNA‐start system) (Figure 3a). In the mRNA‐start system, mRNA is added at a final concentration of 1 µM in the translation reaction mixture, whereas in the DNA‐start system, DNA is used at a much lower concentration (5 nM). Therefore, the mRNA‐start system is more suitable when high library diversity is essential, particularly in the first round of selection. Nevertheless, because the DNA‐start system eliminates multiple preparative steps—including transcription, purification, and annealing—it is attractive for use at later stages to accelerate the selection process. Accordingly, we examined whether display efficiency could be improved in the DNA‐start system.

DNA encoding the wild‐type monobody was added to the translation reaction mixture supplemented with T7 RNA polymerase and incubated at 37°C for 30 min, after which display efficiency was evaluated. Consistent with our expectations, both replacement of the 5‐mer nucleotide at the 5′ end of the PuL from 2′‐OMe RNA to DNA and alteration of its sequence from cccgc to ctcta were effective in improving display efficiency in the DNA‐start system (Figure 3b and Figure 3c). Interestingly, in the DNA‐start system, a marked increase (Figure 3b, Lane 2 vs Lane 4; Figure 3c, 30.8% vs 40.0%) was observed upon replacement of the 5‐mer nucleotide from 2′‐OMe RNA to DNA, compared with the more modest increase (Figure 1c, Lane 4; Figure 1d, 38.4% vs Figure 2e, Lane 2; Figure 2j, 42.8%) observed in the mRNA‐start system. In the mRNA‐start system, the annealing procedure was performed prior to translation. In our validation experiments, both the PuL bearing 2′‐OMe RNA (p‐CCCGC‐t[PuFl]cc) and the PuL bearing DNA (p‐cccgc‐t[PuFl]cc) at the 5′ end formed fully annealed complexes with mRNA‐2 following the annealing procedure (Fig. S11). In contrast, in the DNA‐start system, transcription, annealing, translation, and conjugation occur sequentially without a dedicated annealing step. Therefore, even if the stability of the mRNA/PuL complexes is comparable between the two linkers, differences in annealing kinetics are likely to influence the overall display efficiency.

Notably, p‐ctcta‐t[PuFl]cc exhibited display efficiency comparable to that obtained in the mRNA‐start system (Figure 2f, Lane 2; Figure 2j, 54.3% vs Figure 3b, Lane 6; Figure 3c, 52.2%). Thus, the display efficiency achieved in this experiment is sufficient to allow a transition from the mRNA‐start system in the first round to the DNA‐start system in subsequent rounds.

## 3 Conclusion

Here, we examined various modifications of the mRNA sequence and PuL design to improve the display efficiency of cDNA TRAP display. First, we applied the mRNA sequence previously reported to improve the display efficiency of original TRAP display. Engineering the sequence near the nonsense UAG codon has been proposed to suppress UAG codon misreading, thereby promoting puromycin conjugation to the C terminus of the ALP. As a result of this engineering, we successfully improved the display efficiency of cDNA TRAP display. In addition, the improved display efficiency was comparable to that of the ligation‐containing method.

Second, we identified that the 5‐mer nucleotide at the 5′ end of the PuL affects display efficiency. Replacement of this region from 2′‐OMe RNA to DNA led to slightly increased display efficiency. Furthermore, additional improvement was achieved by altering the sequence of this region to reduce its GC content. Although the detailed mechanism remains unclear, the newly designed PuL and mRNA may prevent secondary structure formation within the single‐stranded oligonucleotides, thereby facilitating hybridization between the PuL and mRNA. Notably, this modification at the 5′ end also improved display efficiency of original TRAP display.

Third, we changed the position at which the puromycin moiety was attached, based on our hypothesis that a shorter distance between the attachment position of the puromycin moiety and the ribosomal A‐site would enhance conjugation and display efficiency. As a result, no improvement in display efficiency was observed, suggesting that the attachment position of the puromycin moiety does not significantly affect display efficiency.

Finally, we evaluated whether display efficiency was improved in the DNA‐start system, which enables more rapid selection than the mRNA‐start system. Modification of the structure and sequence at the 5′ end of the PuL resulted in an improvement in display efficiency similar to the mRNA‐start system, and the improved display efficiency was comparable to that achieved in mRNA‐start system. This result supports a transition from the mRNA‐start system to the DNA‐start system during selection.

This study represents the first optimization of the PuL used in cDNA TRAP display and demonstrates a successful improvement in display efficiency. Because cDNA TRAP display has been limited in practical use by its relatively low display efficiency, the optimization described here could pave the way for broader application of cDNA TRAP display in various selection strategies. Collectively, the improved cDNA TRAP display established in this study is expected to accelerate the discovery of various ALPs that are useful for biological or therapeutic applications.

## Supporting Information

The authors have cited additional references within the Supporting Information [26, 27, 28, 29, 30, 31, 32].

## Conflicts of Interest

The authors declare no conflicts of interest.

## Supporting information

Supplementary Material

## Data Availability

The data that supports the findings of this study are available in the supplementary material of this article.
