# Supplementary material for: Improvement of cDNA TRAP Display via Optimization of Puromycin Linker Design for Enhanced Discovery of Antibody‐Like Proteins
Source: Chembiochem. 2026 May 26;27(10):e70375. doi: 10.1002/cbic.70375 (PMC13206342; doi:10.1002/cbic.70375)
Supplement: Supplementary file 1 — Supplementary Material [file CBIC-27-e70375-s001.pdf]

Supporting Information

Improvement of cDNA TRAP Display via Optimization of Puromycin Linker Design for Enhanced Discovery of Antibody-Like Proteins

Haruto Kosugi,[a] Hiroki Nakanishi,[a] Gosuke Hayashi,[a] Hiroshi Murakami*[a, b, c]

[a] Department of Biomolecular Engineering, Graduate School of Engineering, Nagoya University, Nagoya, Japan
[b] Institute of Nano-Life-Systems, Institutes of Innovation for Future Society, Nagoya University, Nagoya, Japan
[c] Research Institute for Quantum and Chemical Innovation, Institutes of Innovation for Future Society, Nagoya University, Nagoya, Japan

* E-mail: murah@chembio.nagoya-u.ac.jp

Table of Contents

1 Experimental Section 3

1.1 Materials

1.2 Synthesis of the puromycin linker

1.3 Preparation of DNA and mRNA encoding the wild-type monobody

1.4 Preparation of mRNA/PuL or mRNA–PuL complexes

1.5 Analysis of display efficiency

1.6 Pulldown-based evaluation of display efficiency

2 Supporting Tables........ 6

3 Supporting Figures........ 11

4 References........ 22

1 Experimental Section

1.1 Materials

The annealing oligonucleotides and the puromycin linker for original TRAP display were purchased from Japan Bio Services Co., Ltd. (Japan). The puromycin-oligonucleotides were purchased from Tsukuba Oligo Service Co., Ltd. (Japan), and the remaining oligonucleotides were purchased from Fasmac Co., Ltd. (Japan). The sequences of the oligonucleotides are listed in Supporting Table 1. EMCS (N-(6-maleimidocaproyloxy) sulfosuccinimide) was purchased from Dojindo Laboratories (Japan). T4 RNA ligase 1 was purchased from New England Biolabs Japan Inc. (Japan). The composition of the reconstituted cell-free translation system is shown in Supporting Table 2. Most components of the reconstituted cell-free translation system, including T7 RNA polymerase, were prepared using similar procedures described in previous reports.[1–3] Preparation of adenylate kinase, inorganic pyrophosphatase, *Pfu-S* DNA polymerase, and *MMLV* reverse transcriptase were described in a previous report.[4] Preparation of biotin-Phe-$tRNA^{fMet}_{CAU}$ was prepared as previously described.[5–8]

1.2 Synthesis of the puromycin linker

Annealing oligonucleotide (400–800 µM final concentration) was incubated with EMCS (10 mM final concentration) dissolved in DMSO at 37 °C for 2 h in 100 mM HEPES-K (pH 7.8). Q Sepharose™ Fast Flow resin (5 µL; Cytiva) was loaded into a spin column and equilibrated with wash buffer A (5 mM HEPES-K (pH 7.8), 10 mM NaCl, 50% (v/v) DMSO) by rotation at 25 °C for 10 min, followed by centrifugation. EMCS-modified annealing oligonucleotide was added to the column and incubated at 25 °C for 1 h with rotation. After centrifugation, the flow-through was collected. The resin was washed four times with wash buffer A, each wash consisting of incubation at room temperature for 2 min followed by centrifugation. The column was then washed once with wash buffer B (10 mM HEPES-K (pH 7.8), 200 mM NaCl) using the same procedure. Bound oligonucleotides were eluted with elution buffer (10 mM HEPES-K (pH 7.8), 1 M NaCl) by incubation at 42 °C for 10 min, followed by centrifugation. The concentration of the purified EMCS-modified annealing oligonucleotide was determined by measuring absorbance at 260 nm.

The puromycin-oligonucleotide was stored in the presence of TCEP (1 mM final concentration) to maintain a reduced state. After gel filtration using Bio-Gel P-2 Gel (100 µL; Bio-Rad), the concentration of the puromycin-oligonucleotide purified by gel filtration was determined by measuring absorbance at 260 nm.

The EMCS-modified annealing oligonucleotide (180 µM final concentration) and the puromycin-oligonucleotide (45 µM final concentration) were incubated in 100 mM HEPES-K (pH 7.8) at 25 °C for 2 h. The reaction mixture was purified by RP-HPLC using a YMC-Triart Bio C4 column (YMC) (Fig. S7). Elution was performed with a linear gradient of 15–30% acetonitrile in 0.1 M TEAA (triethylammonium acetate) over 30 min at a flow rate of 0.1 mL/min. Oligonucleotides were detected by UV absorbance at 260 nm. The fraction containing the puromycin linker was collected, diluted 10-fold with water, and lyophilized. The lyophilized product was reconstituted in water, and its concentration was determined by measuring absorbance at 260 nm. The calculated yields are summarized in Supporting Table 3. Yields for each linker were calculated by dividing the amount of product obtained after HPLC purification and lyophilization by the initial amount of puromycin-oligonucleotide used in the reaction.

Purified EMCS-modified annealing oligonucleotide, puromycin-oligonucleotide, and puromycin linker (crude and purified samples) were diluted to 0.2 μM in formamide and analyzed by 18% denaturing PAGE containing 6 M urea (Fig. S8).

1.3 Preparation of DNA and mRNA encoding the wild-type monobody

DNA templates encoding the wild-type monobody were prepared by PCR amplification of the WT template using T7SD8M2.F44 and one of the following oligonucleotides: G5S-4Gan21-3.R42, G5R-T-an21-3.R43, G5R-T-an16-3A5.R43, or G5R-T-an16-tagag.R43 as primers. The PCR was carried out under the following conditions: 10 mM Tris-HCl (pH 8.4), 100 mM KCl, 0.1% (v/v) Triton X-100, 2 mM $MgSO_4$, 0.2 mM each dNTP, 2% (v/v) DMSO, 0.375 μM each primer, 0.04 ng/μL WT template, and 2 nM *Pfu-S* DNA polymerase (15 cycles). PCR products were purified using a silica-based method [9] and dissolved in 10 mM Tris-AcOH (pH 7.8). DNA concentrations were determined by 8% PAGE using ExcelBand 100 bp DNA Ladder (Smobio Technology) as a marker.

DNA templates were transcribed by in vitro run-off transcription under the following conditions: 40 mM Tris-HCl (pH 8.4), 1 mM spermidine, 0.01% (v/v) Triton X-100, 10 mM DTT, 25 mM $MgCl_2$, 0.03 N KOH, 5 mM each NTP, and 0.5 μM T7 RNA polymerase. After incubation at 37 °C overnight, the mRNA was purified using a silica-based method. The concentrations of the purified mRNA were determined by measuring absorbance at 260 nm.

1.4 Preparation of mRNA/PuL or mRNA–PuL complexes

The puromycin linker (5 μM final concentration) was annealed to the mRNA encoding the wild-type monobody (5 μM final concentration) in annealing buffer (25 mM HEPES-K (pH 7.8), 200 mM AcOK) by heating at 95 °C for 2 min, followed by cooling to 25 °C and incubation for 5 min.

The mRNA–PuL complex was obtained by ligation of the mRNA/PuL complex. Ligation was carried out under the following conditions: 100 mM HEPES-K (pH 7.8), 40 mM $MgCl_2$, 40% (v/v) DMSO, 0.2% (w/v) PEG6000, 50 μM ATP, 20 mM DTT, and 5% (v/v) T4 RNA ligase 1. After incubation at 25 °C for 1 h, the mRNA–PuL complex was purified using a silica-based method.

1.5 Analysis of display efficiency

The mRNA/PuL or mRNA–PuL (1 μM final concentration) was added to the cell-free translation system, and the reaction mixtures were incubated at 37 °C for 30 min. 1 μL of the reaction mixture was mixed with 11 μL of gel shift buffer (20% (v/v) glycerol, 62.5 mM Tris-HCl (pH 6.8), 5 mM DTT, 10 mM $MgCl_2$, 0.05% (v/v) SDS) and loaded onto urea SDS-PAGE (6 M urea, 0.075% (v/v) SDS, 8% acrylamide/bis mixed solution (37.5:1)). The gel was analyzed using ChemiDoc™ MP Imaging System (Bio-Rad).

In the DNA-start system, DNA encoding the wild-type monobody (5 nM final concentration) and the puromycin linker (1 μM final concentration) were added to the cell-free translation system supplemented with T7 RNA polymerase (1 μM final concentration), and the display efficiency was analyzed using the same procedure described above.

1.6 Pulldown-based evaluation of display efficiency

The PuL/mRNA complex (1 μM final concentration) was added to the cell-free translation system (4 μL) containing 20 μM biotin-Phe-$tRNA^{fMet}_{CAU}$ instead of formyl donor. 1 μL of the reaction mixture was taken before and after translation (37 °C, 30 min) and mixed with 11 μL of gel shift buffer. The remaining 3 μL was mixed with 0.77 μL of 88.2 mM EDTA and 1.89 μL of RT mix [150 mM Tris-HCl (pH 8.4), 225 mM KCl, 75 mM $MgCl_2$, 16.5 mM DTT, 1.5 mM each dNTP, 3.4 μM *MMLV* reverse transcriptase]. Reverse transcription was performed at 42 °C for 30 min. After reverse transcription, 1 μL of the reaction mixture was mixed with 5.36 μL of gel shift buffer and analyzed by urea SDS-PAGE together with the pre- and post-translation samples.

The remaining reverse transcription reaction was diluted 50-fold with HBSTP buffer [50 mM HEPES-K (pH 7.5), 300 mM NaCl, 0.05% (v/v) Tween 20, 0.1% (w/v) PEG 6000]. 10 μL of the diluted sample was incubated with 20 μL of Dynabeads™ M-280 streptavidin (Thermo Fisher Scientific) for 5 min at room temperature. The beads were washed twice with 50 μL of HBSTP buffer for 1 min each. The washed beads were suspended in 100 μL of 1 × PCR dNTPs [10 mM Tris-HCl (pH 8.4), 100 mM KCl, 0.1% (v/v) Triton X-100, 2 mM $MgSO_4$, 0.22 mM each dNTP], followed by heating at 95 °C for 5 min to elute cDNA.

Input samples were prepared by diluting the reverse transcription reaction 5,000-fold with 1 × PCR dNTPs, whereas displayed samples were prepared by diluting the eluted cDNA 10-fold in the same buffer. cDNA recovery was quantified by SYBR Green-based real-time PCR using T7SD8M2.F44 and FN3L1p.R20 as primers.

2 Supporting Tables

Supporting Table 1. Oligonucleotides sequences used in this research. mU, mG and mC represent 2'-OMe RNA. (T) and (C) represent amino-modified C6 dT and dC, respectively. T* represents fluorescein-dT. Abbreviations: SPC18, spacer 18; Hex, hexachloro-fluorescein.

Category	Name	Sequence (5' to 3')	Note
Annealing oligonucleotide	Fl-CCCGC-tcc	mCmCmCmGmCmCmUmCmGmCmGmCmCmCmGmC mCmG(T)CC	5'-Fluorescein
	p-CCCGC-tcc	mCmCmCmGmCmCmUmCmGmCmGmCmCmCmGmC mCmG(T)CC	5'-Phosphate
	p-cccgc-tcc	CCCGCmCmUmCmGmCmGmCmCmCmGmCmCmG(T) CC	5'-Phosphate
	p-ctcta-tcc	CTCTAmCmUmCmGmCmGmCmCmCmGmCmCmG(T) CC	5'-Phosphate
	p-ctcta-Ucc (C2am6)	CTCTAmCmUmCmGmCmGmCmCmCmGmCmCmGmU (C)C	5'-Phosphate
	p-ctcta-Ucc (C1am6)	CTCTAmCmUmCmGmCmGmCmCmCmGmCmCmGmU C(C)	5'-Phosphate
Puromycin-oligonucleotide	HS-Pu	$(SPC18)_2$CC	5'-Thiol C6, 3'-Puromycin
	HS-PuFl	(SPC18)T*(SPC18)CC	5'-Thiol C6, 3'-Puromycin
Puromycin linker (Original TRAP)	Hex-CCCGC-Pu	mCmCmCmGmCmCmUmCmGmCmGmCmCmCmGmC mCmGmUmCmC$(SPC18)_5$CC	5'-Hex, 3'-Puromycin
	p-cccgc-PuFl	CCCGCmCmUmCmGmCmGmCmCmCmGmCmCmGm UmCmC$(SPC18)_3$T*$(SPC18)_2$CC	5'-Phosphate, 3'-Puromycin
	p-ctcta-PuFl	CTCTAmCmUmCmGmCmGmCmCmCmGmCmCmG mUmCmC$(SPC18)_3$T*$(SPC18)_2$CC	5'-Phosphate, 3'-Puromycin
Template	WT template	ATACTAATACGACTCACTATAGGATTAAGGAGGTGATA TTTATGCAAGCCAATTCTGGTTCTCTGGAAGTTGTGG AAGCCAGCCCGACGAGCATTCAGATTTCTTGGGACG CTCCGGCGGTCACGGTTCGCTACTATCGCATTACCTA TGGCGAAACCGGCGGTAACAGTCCGGTCCAGGAATT TACGGTGCCGGGTTCAAAATCGACCGCGACGATTTC CGGCCTGAAACCGGGTGTTGATTATACCATCACGGTG TACGCAGTTACCGGTCGTGGTGACAGCCCGGCCAGC TCTAAACCGATTTCTATCAACTACCGCACGGGTGGAG GAGGAGGTAGCT	

Primer	T7SD8M2.F44	ATACTAATACGACTCACTATAGGATTAAGGAGGTGATA TTTATG
	G5S-4Gan21-3.R42	CCCGCCTCGCGCCCGCCGTCCCTAGCTACCTCCTCC TCCACC
	G5R-T-an21-3.R43	CCCGCCTCGCGCCCGCCGTCCACTATCGGCCTCCTC CTCCACC
	G5R-T-an16-3A5.R43	TTTTTCTCGCGCCCGCCGTCCACTATCGGCCTCCTCC TCCACC
	G5R-T-an16-tagag.R43	CTCTACTCGCGCCCGCCGTCCACTATCGGCCTCCTC CTCCACC
	FN3L1p.R20	CCAAGAAATCTGAATGCTCG

Supporting Table 2. Composition of the reconstituted cell-free translation system used in this research.

Components	Final concentrations
ATP	2 mM
GTP	2 mM
CTP	1 mM
UTP	1 mM
Creatine Phosphate	20 mM
HEPES-K (pH 7.6)	50 mM
Potassium Acetate	100 mM
Magnesium Acetate	12 mM
Spermidine	2 mM
DTT	1 mM
10-HCO-H4folate	0.1 mM
E. coli tRNA mix	1.5 mg/mL
AlaRS	0.73 μM
ArgRS	0.03 μM
AsnRS	0.38 μM
AspRS	0.13 μM
CysRS	0.02 μM
GlnRS	0.06 μM
GluRS	0.23 μM
GlyRS	0.09 μM
HisRS	0.02 μM
IleRS	0.4 μM
LeuRS	0.04 μM
LysRS	0.11 μM
MetRS	0.03 μM
PheRS	0.68 μM
ProRS	0.16 μM
SerRS	0.04 μM
ThrRS	0.09 μM
TrpRS	0.03 μM
TyrRS	0.02 μM
ValRS	0.02 μM
Methionyl-tRNA formyltransferase	0.6 μM
Elongation factor Tu/Ts	20 μM

Elongation factor G	0.26 μM
Initiation factor 1	2.7 μM
Initiation factor 2	0.4 μM
Initiation factor 3	1.5 μM
Release factor 2	0.25 μM
Release factor 3	0.17 μM
Ribosome recycling factor	0.5 μM
Creatine kinase	4 μg/mL
Adenosine kinase	0.1 μM
Inorganic pyrophosphatase	0.1 μM
Nucleoside-diphosphate kinase	0.1 μM
Ribosome	1.2 μM

Supporting Table 3. Calculated yields of puromycin linkers used in this study. Puromycin linkers were synthesized using annealing oligonucleotides (final concentration: 180 μM) and puromycin–oligonucleotides (final concentration: 45 μM). Yields for each linker were calculated by dividing the amount of product obtained after HPLC purification and lyophilization by the initial amount of puromycin-oligonucleotide used in the reaction.

Name	Reaction volume (μL)	Reconstitution volume (μL)	Concentration (μM)	Calculated yield (%)
Fl-CCCGC-t[Pu]cc	30.0	22.0	26.7	43.5
p-CCCGC-t[PuFl]cc	50.0	25.6	34.8	39.6
p-cccgc-t[PuFl]cc	50.0	19.0	39.9	33.7
p-ctcta-t[PuFl]cc	50.0	18.8	36.1	30.2
p-ctcta-Uc[PuFl]c	20.0	28.8	19.5	62.3
p-ctcta-Ucc[PuFl]	40.0	20.0	46.1	51.2

3 Supporting Figures

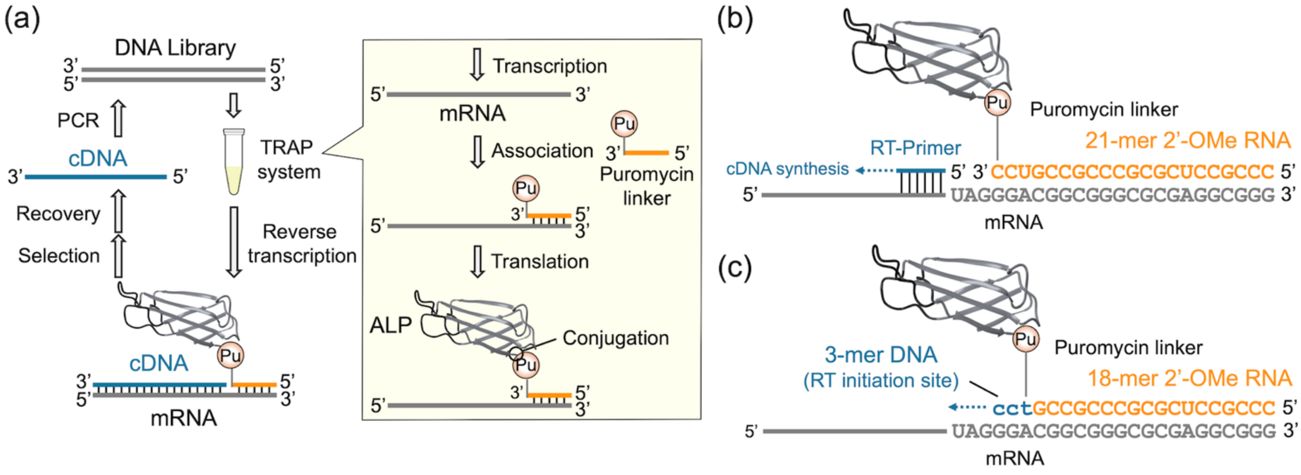

Supporting Figure 1. TRAP display and puromycin linkers used in original TRAP and cDNA TRAP display. (a) Schematic representation of TRAP display. By addition of DNA to the TRAP system, sequential reactions—including transcription of DNA into mRNA, association of a PuL, translation of mRNA into ALPs, and conjugation between the ALP and PuL—proceed continuously. After reverse transcription (RT) and selection, recovered cDNAs were amplified by PCR. (b) Design of a PuL in original TRAP display. The PuL is synthesized using 2'-OMe RNA to prevent promoter-independent transcription mediated by T7 RNA polymerase,[4] and RT is initiated upon addition of a primer. (c) Design of a PuL in cDNA TRAP display. The 3-mer nucleotide at the 3' end is replaced with DNA, and RT is initiated from this region.

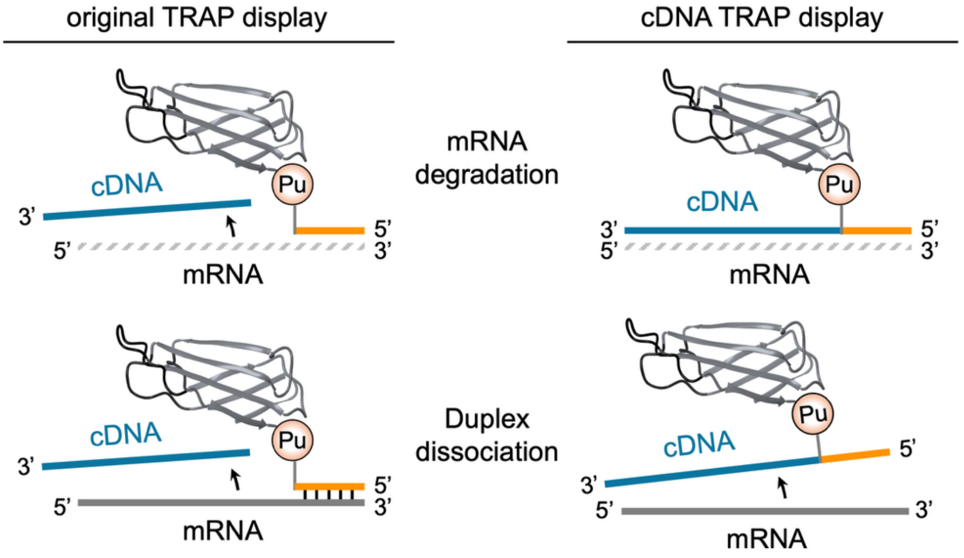

Supporting Figure 2. Comparison of original TRAP display and cDNA TRAP display. In original TRAP display, degradation of mRNA or dissociation of cDNA from mRNA can disrupt the linkage between an ALP and its encoding gene. In contrast, in cDNA TRAP display, the cDNA is covalently linked to the ALP–PuL conjugate, ensuring preservation of the genetic information of the ALP under these conditions.

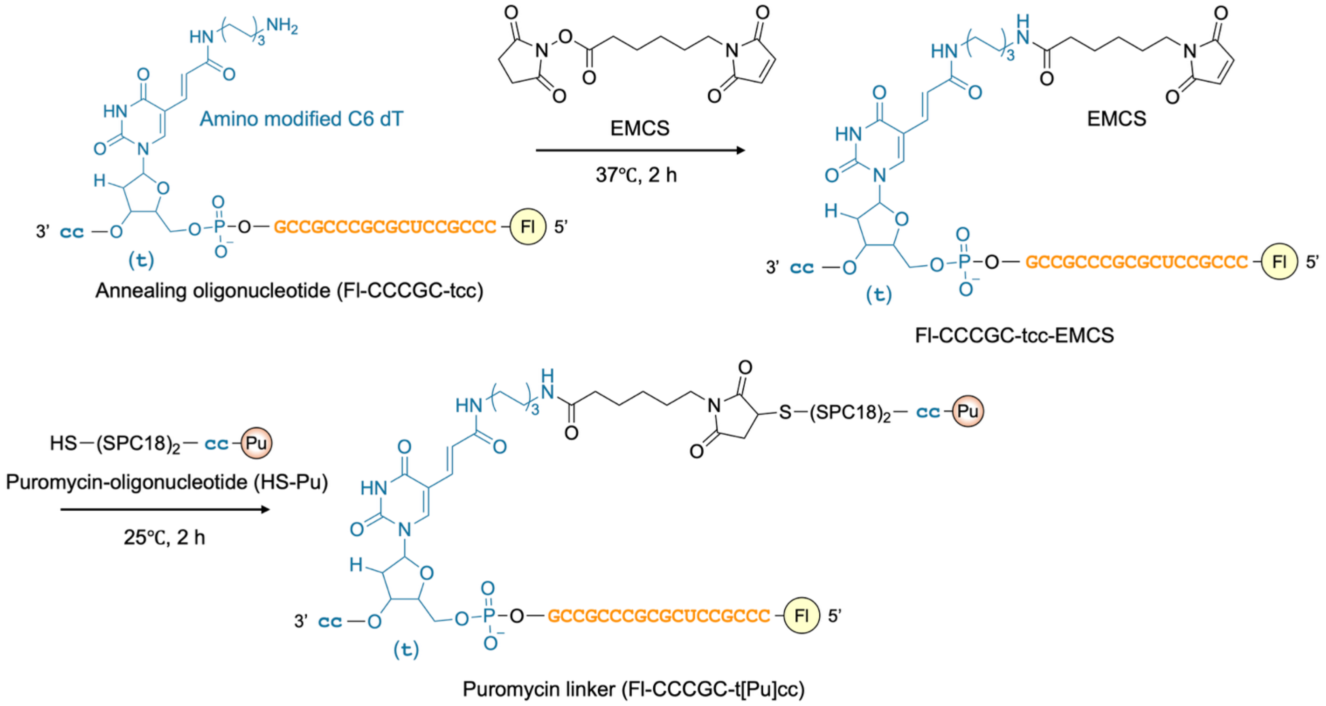

Supporting Figure 3. Synthesis scheme of Fl-CCCGC-t[PuFl]cc. The PuL was synthesized by reacting the amino-modified C6 dT within an annealing oligonucleotide with EMCS (N-(6-maleimidocaproyloxy) sulfosuccinimide), followed by coupling with a puromycin-oligonucleotide bearing a reduced thiol group. DNA and 2'-OMe RNA are shown in blue and orange, respectively. SPC18 represents spacer 18.

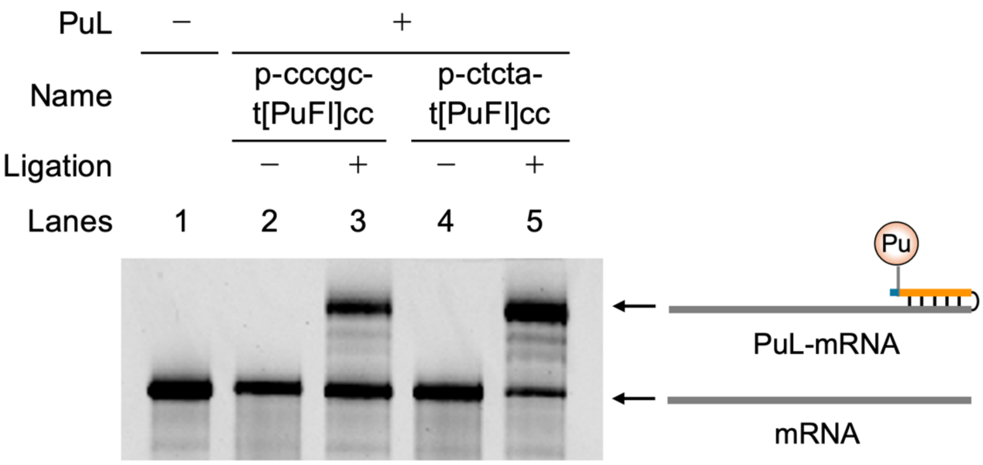

Supporting Figure 4. Urea denaturing PAGE analysis of ligated complexes between p-cccgc-t[PuFl]cc or p-ctcta-t[PuFl]cc and mRNA-3. Ligation(+) indicates samples incubated in the presence of T4 RNA ligase, whereas Ligation(-) indicates samples incubated for the same duration in the absence of the enzyme.

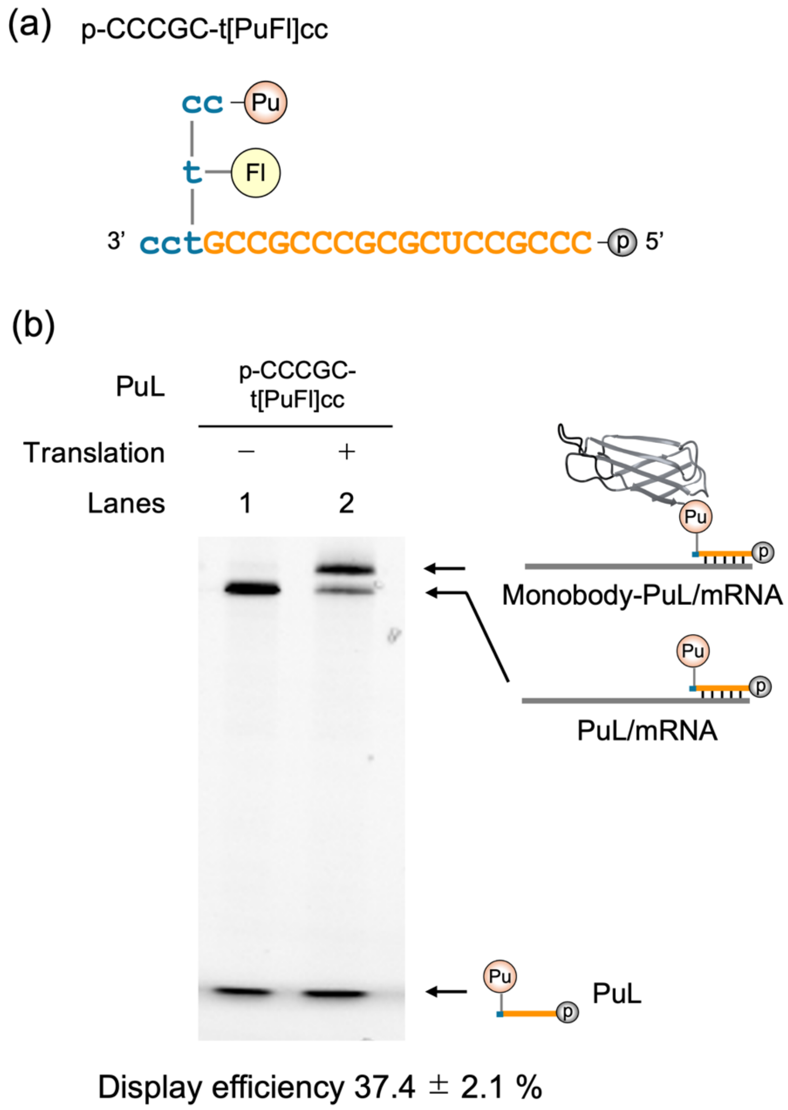

Supporting Figure 5. Display efficiency of cDNA TRAP display using p-CCCGC-t[PuFl]cc. (a) Structure of p-CCCGC-t[PuFl]cc. Color coding is the same as in Fig. S3. (b) Urea SDS-PAGE analysis of display efficiency using p-CCCGC-t[PuFl]cc. The display efficiency is presented as the mean ± standard deviation (n=3).

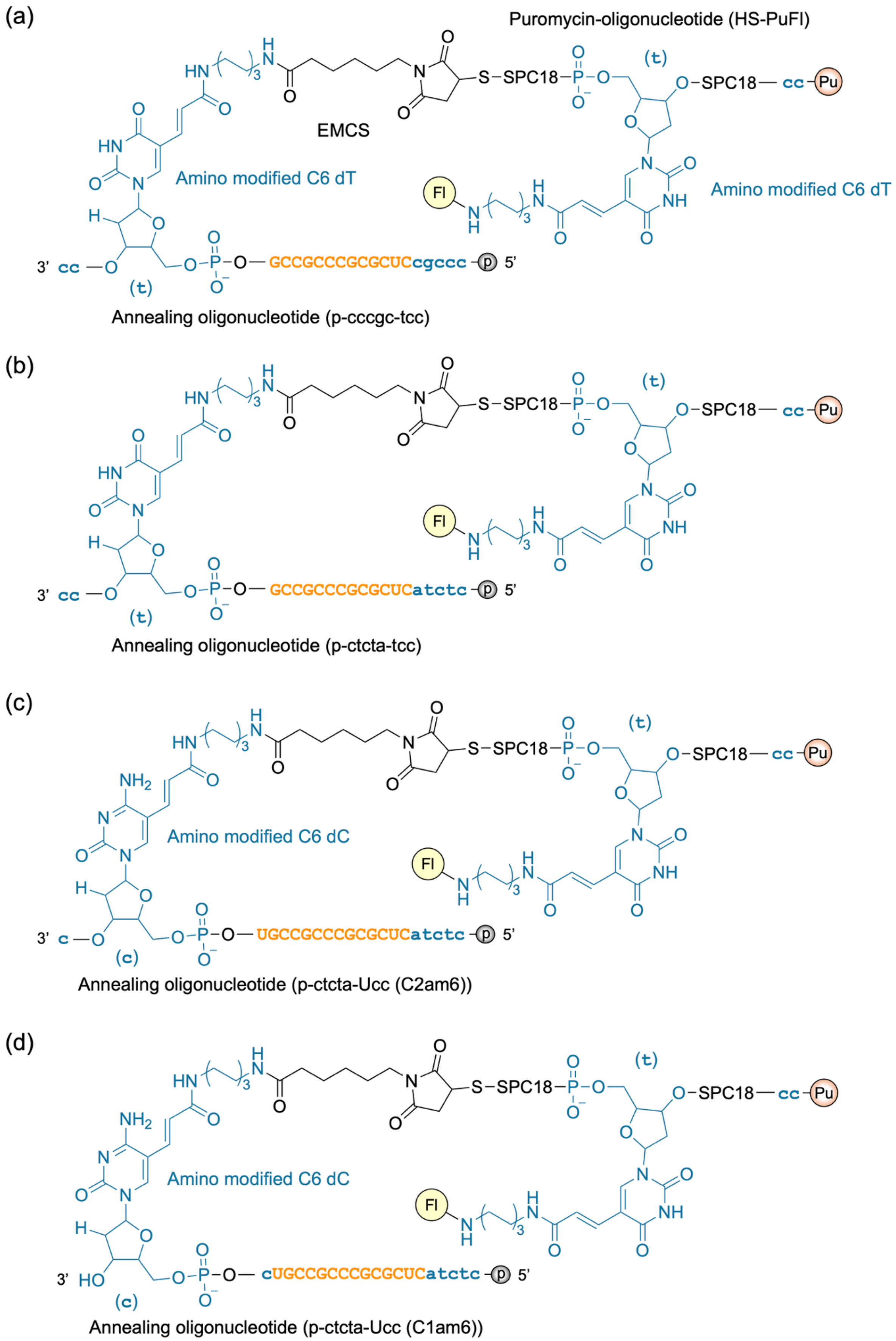

Supporting Figure 6. Detailed structures of PuLs used in this study: (a) p-cccgc-t[PuFl]cc; (b) p-ctcta-t[PuFl]cc; (c) p-ctcta-Uc[PuFl]c; (d) p-ctcta-Ucc[PuFl]. Color coding is the same as in Fig. S3.

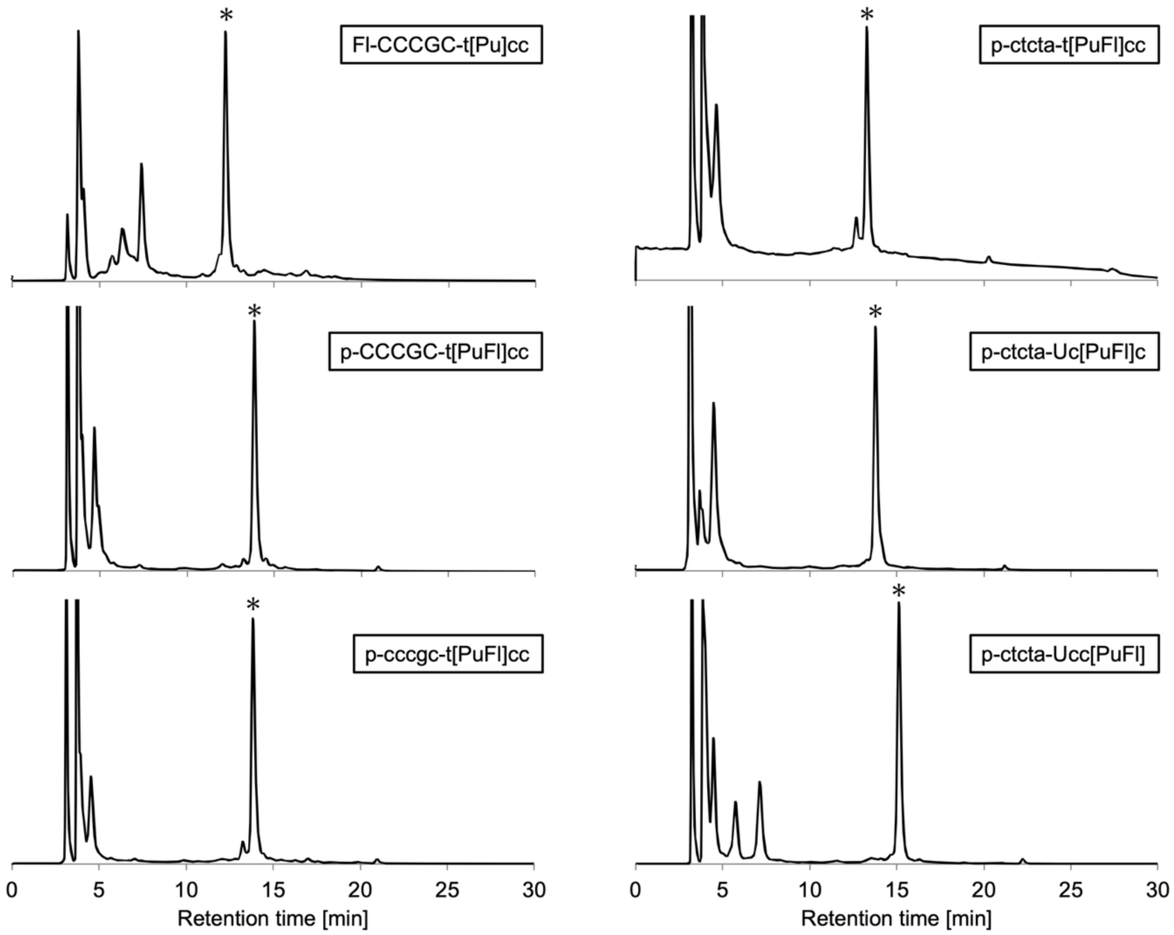

Supporting Figure 7. HPLC chromatograms for purification of synthesized PuLs. HPLC was performed using a linear gradient of 15–30% acetonitrile in 0.1 M TEAA (triethylammonium acetate) over 30 min. The fraction corresponding to the peak marked with an asterisk (∗) was collected.

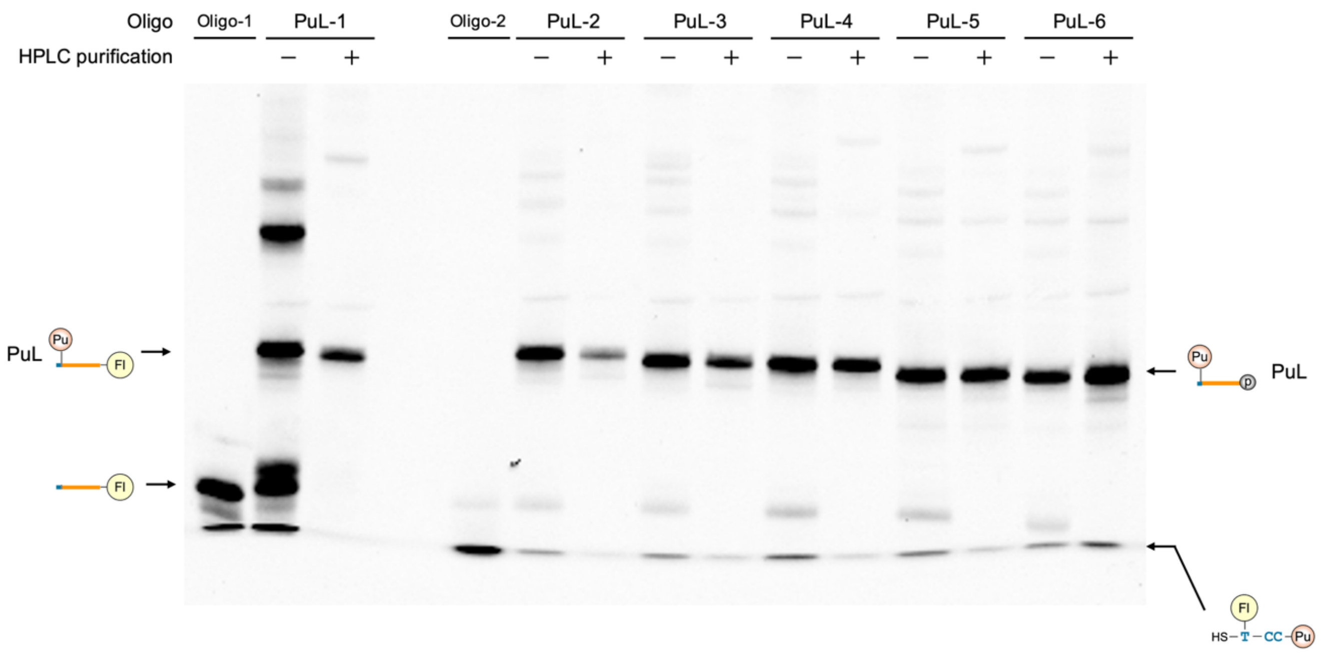

Supporting Figure 8. Urea denaturing PAGE analysis of synthesized PuLs before and after HPLC purification. Oligo-1 and Oligo-2 represent Fl-CCCGC-tcc and HS-PuFl, respectively. PuL-1–6 represent Fl-CCCGC-t[Pu]cc, p-CCCGC-t[PuFl]cc, p-cccgc-t[PuFl]cc, p-ctcta-t[PuFl]cc, p-ctcta-Uc[PuFl]c, and p-ctcta-Ucc[PuFl], respectively.

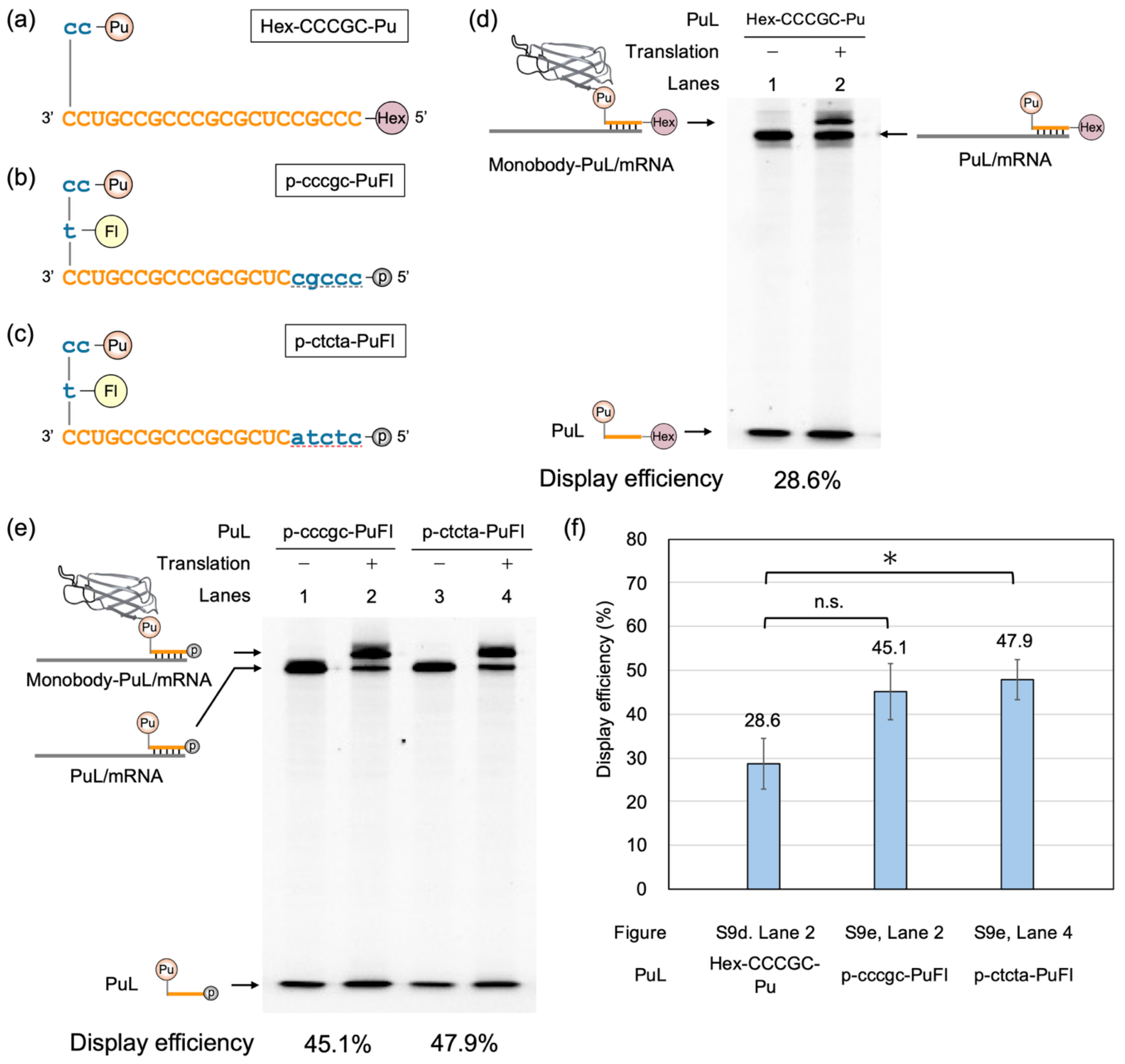

Supporting Figure 9. Improvement of display efficiency in original TRAP display. (a), (b), (c) Structures of Hex-CCCGC-Pu, p-cccgc-PuFl, and p-ctcta-PuFl, respectively. Color coding is the same as in Fig. S3. Hex-CCCGC-Pu was also used in a previous study.[4] (d) Urea SDS-PAGE analysis of display efficiency using Hex-CCCGC-Pu. (e) Urea SDS-PAGE analysis of display efficiencies using p-cccgc-PuFl and p-ctcta-PuFl. (f) Quantification of display efficiency based on the urea SDS-PAGE analyses shown in Fig. S9d and Fig. S9e. Error bars represent the standard deviation of triplicate experiments.

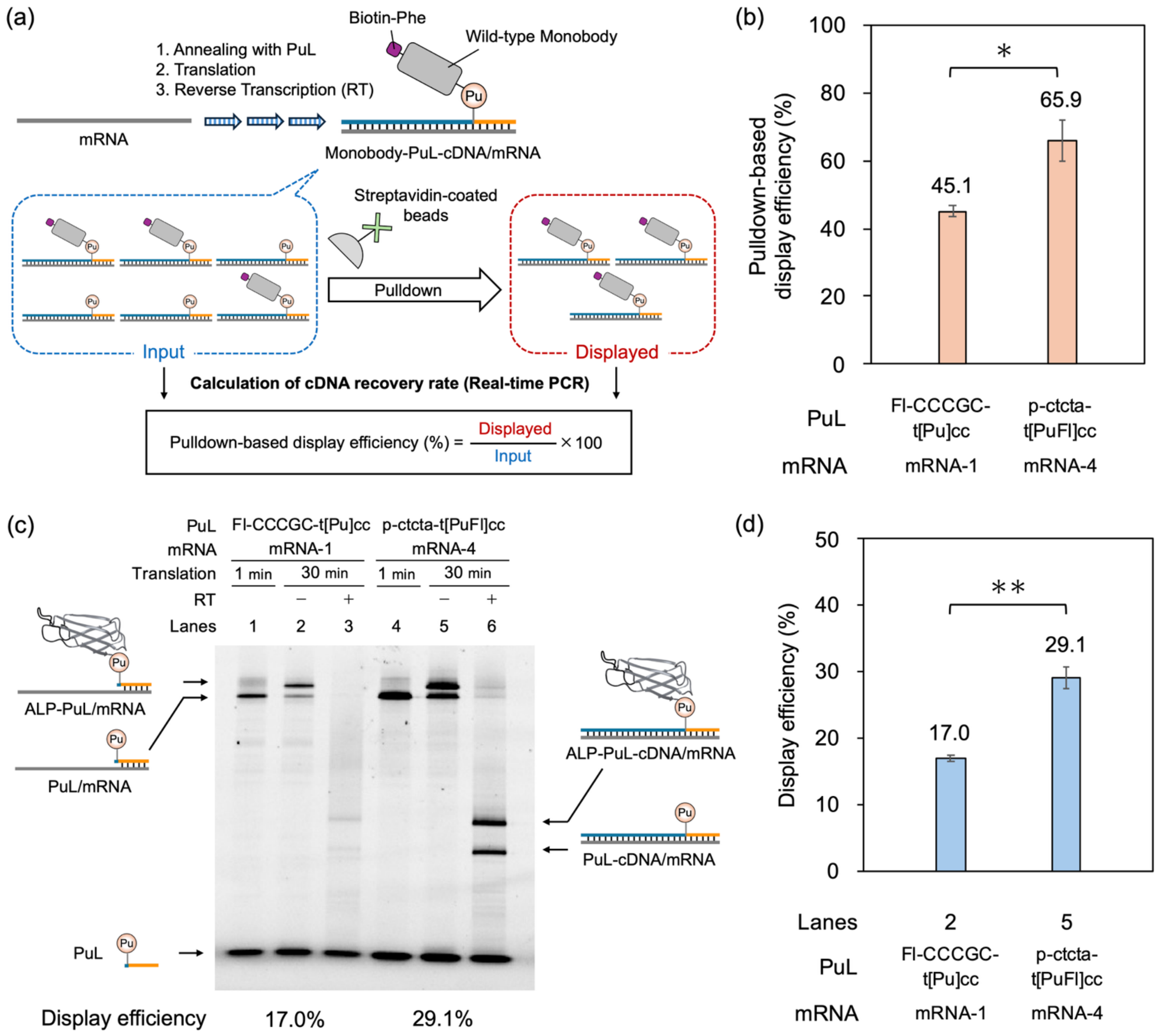

Supporting Figure 10. Pulldown-based evaluation of display efficiency. (a) Schematic representation of pulldown-based assay. Biotin-Phe was introduced to the start codon using the Flexizyme system, and the displayed monobody was subsequently isolated by pulldown. The amount of recovered cDNA was then quantified by real-time PCR to evaluate the formation efficiency of the cDNA-monobody complex. (b) Quantification of display efficiency based on the pulldown assay. Error bars represent the standard deviation of triplicate experiments. (c) Urea SDS-PAGE analysis of display efficiencies. (d) Quantification of display efficiency based on the urea SDS-PAGE analysis shown in Fig. S10c. Error bars represent the standard deviation of triplicate experiments.

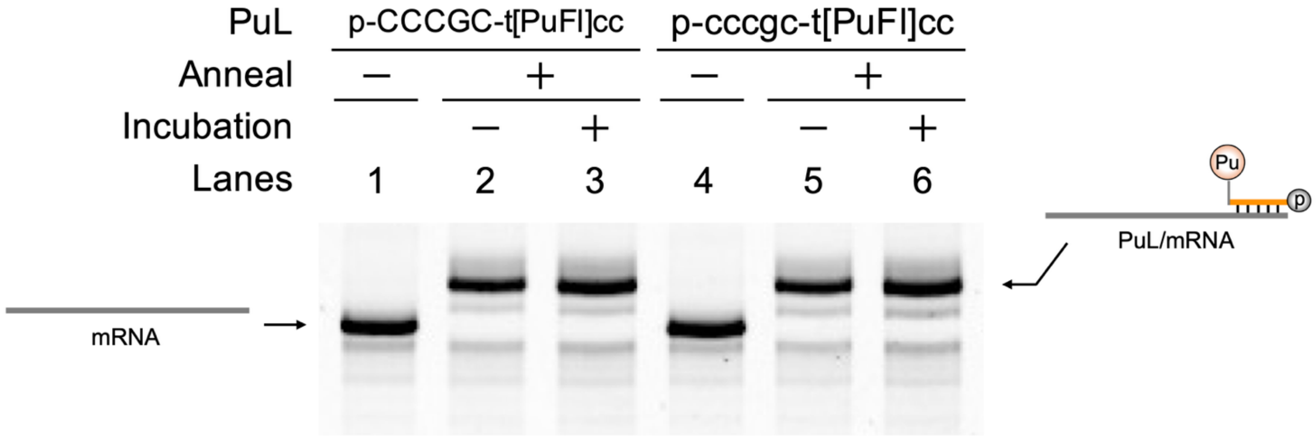

Supporting Figure 11. Evaluation of annealing efficiency of p-CCCGC-t[PuFl]cc and p-cccgc-t[PuFl]cc, and the stability of the mRNA/PuL complexes. Anneal (−) indicates mRNA alone, whereas Anneal (+) indicates samples subjected to the same annealing conditions used in this study. After dilution with water to a final concentration of 1 μM, samples were incubated at 37 °C for 30 min. Samples were then diluted in formamide and analyzed by 6 M urea-PAGE. To detect non-fluorescently labeled mRNA, ethidium bromide staining was used.

4 References

[1] Y. Shimizu, A. Inoue, Y. Tomari, T. Suzuki, T. Yokogawa, K. Nishikawa, T. Ueda, “Cell-free translation reconstituted with purified components” *Nat. Biotechnol.* **2001**, *19*, 751–755.

[2] P. C. Reid, Y. Goto, T. Katoh, H. Suga, “Charging of tRNAs using ribozymes and selection of cyclic peptides containing thioesters” *Methods Mol. Biol.* **2012**, *805*, 335–348.

[3] H. Ohashi, Y. Shimizu, B. W. Ying, T. Ueda, “Efficient protein selection based on ribosome display system with purified components” *Biochem. Biophys. Res. Commun.* **2007**, *352*, 270–276.

[4] T. Kondo, Y. Iwatani, K. Matsuoka, T. Fujino, S. Umemoto, Y. Yokomaku, K. Ishizaki, S. Kito, T. Sezaki, G. Hayashi, H. Murakami, “Antibody-like proteins that capture and neutralize SARS-CoV-2” *Sci. Adv*. **2020**, *6*, eabd3916.

[5] T. Ishizawa, T. Kawakami, P. C. Reid, H. Murakami, “TRAP display: A high-speed selection method for the generation of functional polypeptides” *J. Am. Chem. Soc*. **2013**, *135*, 5433–5440.

[6] H. Murakami, A. Ohta, H. Ashigai, H. Suga, “A highly flexible tRNA acylation method for non-natural polypeptide synthesis” *Nat. Methods*, **2006**, *3*, 357–359.

[7] H. Suga, P. A. Lohse, J. W. Szostak, “Structural and kinetic characterization of an acyl transferase ribozyme” *J. Am. Chem. Soc.* **1998**, *120*, 1151–1156.

[8] H. Saito, D. Kourouklis, H. Suga, “An in vitro evolved precursor tRNA with aminoacylation activity” *EMBO J.* **2001**, *20*, 1797–1806.

[9] J. S. Boyle, A. M. Lew, “An inexpensive alternative to glassmilk for DNA purification” *Trends Genet*. **1995**, *11*, 8.
